# Protected Blend of Organic Acids and Essential Oils Improves Growth Performance, Nutrient Digestibility, and Intestinal Health of Broiler Chickens Undergoing an Intestinal Challenge

**DOI:** 10.3389/fvets.2019.00491

**Published:** 2020-01-10

**Authors:** Catarina Stefanello, Daniele P. Rosa, Yuri K. Dalmoro, Ana L. Segatto, Marcia S. Vieira, Mariana L. Moraes, Elizabeth Santin

**Affiliations:** ^1^Department of Animal Science, Federal University of Santa Maria, Santa Maria, Brazil; ^2^Department of Biochemistry and Molecular Biology, Federal University of Santa Maria, Santa Maria, Brazil; ^3^Jefo Nutrition Inc., Saint-Hyacinthe, QC, Canada

**Keywords:** antibiotic, broiler, essential oil, intestinal health, organic acid

## Abstract

The growing restriction of antibiotic growth promoters (AGP) use in farming animals has raised a concern regarding the viability of the animal production system. In this new context, feed additives with proven positive impact on intestinal health may be used as strategy to avoid losses on performance. The aim of this study was to evaluate the effects of a protected blend of organic acids and essential oils [P(OA+EO)] on growth performance, nutrient digestibility, and intestinal health of broiler chickens. A total of 1,080 Cobb × Cobb 500 male broilers were randomly distributed in four treatments with 10 replicates (27 birds/each). Treatments were as follow: non-challenged control; challenged control; AGP (enramycin at 10 g/t); and P(OA+EO) at 300 g/t. All birds on challenged groups were challenged with *Eimeria* spp. at 1 day and with *Clostridium perfringens* at 11, 12, and 13 days. Body weight gain (BWG), feed intake and feed conversion ratio (FCR) were evaluated until 42 days. At 17 days, one bird per pen was orally gavaged with fluorescein isothiocyanate-dextran (FITC-d) and blood samples were collected for FITC-d detection to assess intestinal permeability. At 21 days, apparent ileal nutrient and energy (IDE) digestibility, intestinal macroscopic and histologic alterations (ISI) and, expression of mucin2 (MUC2), claudin1 (CLDN1), and occludin (OCLN) genes in the jejunum were evaluated. From 1 to 42 days, birds from the non-challenged and P(OA+EO) groups had greater (*P* < 0.001) BWG compared to challenged control and AGP groups. The challenged control group presented the worst FCR (*P* < 0.001). IDE was 106 kcal/kg greater when broilers were fed P(OA+EO) compared to the challenged control group. Broilers supplemented with P(OA+EO) had improved intestinal integrity with lower blood FITC-d concentration and ISI scores, and greater expression of MUC2, CLDN1, and OCLN genes compared to the challenged control group (*P* < 0.05). In conclusion, the P(OA+EO) and the AGP led to increased growth performance, nutrient digestibility and intestinal health of challenged broilers. A marked difference occurred in favor of the P(OA+EO), suggesting that this blend may be used to improve intestinal health and broiler growth performance in AGP free programs.

## Introduction

The intestinal health of broiler chickens has been related to different factors such as microbiota balance, enteric pathogens, excess of nutrients, water quality, management, and biosecurity (1). Disruptions in the gastrointestinal tract (GIT) can affect digestive, absorptive, metabolic, and immunological functions in birds (2). Therefore, a disturbance in the intestinal homeostasis may result in economic losses, mainly due to poor growth performance and treatment intervention costs (3).

A common alternative to improve birds' intestinal health has been to formulate diets with feed additives. Antibiotic growth promoters (AGP) have been used in poultry production to prevent bacterial infections and to subsequently to promote growth (4). However, there is an increased pressure to remove AGP in poultry production (5, 6). In AGP-free poultry production, natural feed additives with proven positive effects on broiler chickens' intestinal health can play a key role in improving growth performance, especially when combined with complementary biosecurity practices (7).

In order to find substitutes for AGP, different natural additives have been evaluated (8). Blended natural additives aiming to improve intestinal health are commercially available. Although their modes of action have not yet been elucidated, previous studies have shown that they may modulate gut microflora (8). Among alternatives, organic acids (OA) and essential oils (EO) have been used extensively for broiler chickens in different countries (9). Several studies, in which broilers were supplemented with organic acids and essential oils, reported improvement in growth performance and feed efficiency (10, 11).

Organic acids are naturally found in the intestinal tract of animals, being originated from microbial fermentation. They are also distributed in animal and plant tissues. One of the characteristics of these acids is that they do not dissociate completely in water (12) and are related to the inhibition of bacterial growth (13). The EO blends are mixtures of phytochemical compounds that have selective antimicrobial properties (14), acting against *Clostridium perfringens* proliferation and helping to control coccidia infection, reducing necrotic enteritis (10, 14).

The combination of OA and EO is the great interest because it may result in a synergic or additive effect on the intestinal health and growth performance, as observed in previous studies (11). The modulation of the intestinal microbiota may be the main mode of action linked to the synergic effects of a blend of OA and EO. The great hydrophobicity property of the EO increases the bacterial membrane permeability, which may facilitate the influx of OA into the cytoplasm. In its undissociated form, the OA have the ability to reduce the internal pH and disturb the bacterial metabolism (15, 16).

Although there are several studies showing the effects of OA and EO on intestinal health and growth performance of broiler chickens, the product composition in terms of type and quantity of active compounds and offering form may be the reason for the variable results found in the literature. Therefore, the objective of the present study was to evaluate the effects of a protected blend of organic acids and essential oils on growth performance, nutrient digestibility, and intestinal health of broiler chickens undergoing an intestinal challenge while comparing it to an AGP. Our findings may help increasing the knowledge regarding the mode of action of a commercial protected blend of organic acids and essential oils in broiler chickens undergoing an intestinal challenge while investigating it effectiveness for AGP free programs.

## Materials and Methods

All procedures were approved by the Ethics and Research Committee of the Federal University of Santa Maria, Santa Maria, Brazil (number 5404280717) under the supervision of a licensed poultry veterinarian.

### Bird Husbandry

A total of 1,080 1-day-old, slow-feathering Cobb × Cobb 500 male broiler chicks were vaccinated against Marek's disease at the hatchery (Vibra Group, RS, Brazil). Chicks had 43 ± 1 g and were randomly placed into 40 floor pens (1.66 × 1.75 m; 9.3 birds/m^2^; 27 birds per pen). Pens were covered with wood shavings and were equipped with a 15 kg capacity tube feeder and five nipple drinkers in a climate-controlled poultry house. Average temperature was 32°C at placement being reduced by 1°C every 2 days until 23°C to provide comfort throughout the study with the use of thermostatically controlled heaters, fans and foggers. Lighting was continuous until 17 days of age, with a 16 h light and 8 h dark cycle used afterwards. Birds had *ad libitum* access to water and mash feeds.

### Experimental Design, Diets, and Treatments

Birds were distributed in four experimental treatments with 10 replicates in a completely randomized design. A four-phase feeding program was used with pre-starter (1–7 days), starter (7–21 days), grower (21–35 days), and finisher (35–42 days) diets formulated according to Rostagno et al. (17) (Table 1). To determine ileal digestibility, Celite at 1% (Celite, Celite Corp., Lompoc, CA) was used as indigestible marker. The starter diet with the indigestible marker was provided 48 h prior to ileal digesta collection at 21 days.

**Table 1:** Ingredient and nutrient composition of the experimental diets, as fed basis.

**Item**	**Pre-starter (1–7 days)**	**Starter (7–21 days)**	**Grower (21–35 days)**	**Finisher (35–42 days)**
**Ingredient, %**				
Corn	46.64	47.08	50.11	60.68
Soybean meal, 46%	45.18	43.97	40.40	31.50
Soybean oil	4.53	5.59	6.49	5.28
Limestone	1.32	1.21	1.07	0.88
Dicalcium phosphate	1.05	0.84	0.71	0.46
Salt	0.53	0.48	0.48	0.41
Vitamin and mineral premix^a^	0.20	0.18	0.15	0.15
DL-Methionine, 99%	0.34	0.40	0.31	0.26
L-Lysine HCl, 78%	0.15	0.16	0.19	0.26
L-Threonine, 98.5%	0.03	0.05	0.05	0.05
Choline chloride, 60%	0.02	0.03	0.03	0.07
Phytase^b^	0.01	0.01	0.01	0.01
**Nutrient and energy composition, % or as shown**				
Metabolizable energy, kcal/kg	3,000	3,100	3,200	3,250
Crude protein	25.17	24.18	22.52	19.50
Ca	1.01	0.91	0.82	0.66
Available P	0.42	0.38	0.35	0.28
Na	0.23	0.22	0.21	0.20
Choline, mg/kg	1,600	1,600	1,500	1,500
Digestible lysine^c^	1.36	1.31	1.23	1.07
Digestible methionine + Cystine	1.00	0.97	0.91	0.79
Digestible threonine	0.89	0.86	0.82	0.71
Digestible tryptophan	0.27	0.26	0.23	0.19
Digestible arginine	1.46	1.40	1.32	1.16
Digestible valine	1.03	1.01	0.95	0.82
Digestible isoleucine	0.95	0.91	0.85	0.74
Digestible leucine	1.46	1.41	1.36	1.24

a *Composition per kg of feed: vitamin A, 9,000 UI; vitamin D_3_, 2,500 UI; vitamin E, 20 UI; vitamin K_3_, 2,5 mg; thiamine, 2 mg; riboflavin, 6 mg; pyridoxine, 3.8 mg; cyanocobalamin, 0.015 mg, pantothenic acid, 12 mg; niacin, 35 mg; folic acid, 1,5 mg; biotin, 0.1 mg; iron, 40 mg; zinc, 80 mg; manganese, 80 mg; copper, 10 mg; iodine, 0.7 mg; selenium, 0.25 mg*.

b *Ronozyme HiPhos (GT) with 10,000 FYT/g (Novozymes A/S, Bagsvaerd, Denmark)*.

c *Ratios of digestible amino acids to digestible Lys were maintained at TSAA: 0.75; Thr: 0.65; Val: 0.70; Trp: 0.17; Arg: 1.08; Ile: 0.67*.

The four treatments (T) received the same basal corn-soybean meal-based diet. The only difference between treatments was regarding the feed additive supplemented and the presence or not of an intestinal challenge as it follows: T1, non-challenged control; T2, challenged control; T3, challenged and an antibiotic growth promoter (AGP, enramycin, 10 g/t); and T4, challenged and a blend of protected organic acids and essential oils [P(OA+EO), Jefo Nutrition Inc. product, Saint Hyacinthe, Canada, 300 g/t]. The organic acids present in this blend are fumaric, sorbic, malic, and citric acids and the essential oils are thymol, vanillin, and eugenol. Except those on non-challenged control treatment, all birds were challenged at 1 day, via individual oral gavaged with a commercially approved coccidial vaccine, 10× the regular dose (Bio-Coccivet R® live vaccine, containing *Eimeria acervulina, E. brunetti, E. maxima, E. necatrix, E. praecox, E. tenella*, and *E. mitis*; Biovet Vaxxinova, SP, Brazil). At 11, 12, and 13 days, birds were individually orally gavaged with 1 mL/bird of *Clostridium perfringens*. The analyzed concentration was 2.2 × 10^9^; 3 × 10^9^, and 4× 10^9^ cfu/mL at 11, 12, and 13 days, respectively (LABMOR UFPR, PR, Brazil). This intestinal challenge model, created with the objective of generating a dysbiosis, was previously used by the research group (18, 19).

### Experimental Procedures

Chicks were individually weighed in groups of 27 birds per pen before placement. Body weight (BW) averaged by pen was recorded weekly. Body weight gain (BWG), feed intake (FI), and feed conversion ratio corrected for the weight of dead birds (FCR) were determined by feeding phase. Mortality was recorded daily.

On d 17 post-hatch, one bird per pen (with the average BW of the experimental unit; *n* = 40) was orally gavaged with 2.2 mg/bird of systemic fluorescein isothiocyanate-dextran (FITC-d, 3–5 kDa; 4,000 mol weight; Sigma-Aldrich, Brazil) dissolved in one ml Milli-Q water 18.2 MΩ cm at 25°C (20). Blood samples were collected 1 h after gavage. To detect FITC-d level in serum, blood was kept at room temperature for 3 h to allow clotting, and centrifuged (500 × g for 15 min) to separate the serum. Fluorescence levels of diluted serum (1:1 in phosphate buffered saline, PBS) were measured at an excitation wavelength of 485 nm and emission wavelength of 528 nm (Synergy HT, Multi-mode microplate reader, BioTek Instruments, Inc., VT). The FITC-d concentration (μg/mL) of serum was calculated based on a standard curve (21). FITC-d is a molecule with high molecular weight that is only detected in the blood when the intestinal mucosa is damaged, presenting a higher permeability.

On d 21 post-hatch, four birds per pen (*n* = 160) were weighed and euthanized by asphyxiation using carbon dioxide. Ileal digesta was collected from the distal two-thirds of the ileum (portion of the small intestine from Meckel's diverticulum to approximately 1 cm anterior to the ileo-cecal junction) by flushing with distilled water into plastic containers. Samples were pooled by experimental unit, immediately frozen in liquid nitrogen, and stored in a freezer at −20°C until lyophilized (Christ Alpha 2-4 LD Freeze Dryer, Newtown, UK). Diet and freeze-dried samples of ileal digesta were ground to pass a 0.5 mm screen in a grinder (Tecnal, TE-631/2, São Paulo, Brazil). Mucosa samples of one bird per pen (*n* = 40) from the middle of jejunum were scraped into Trizol reagent (Invitrogen, California, USA), immediately frozen in liquid nitrogen, and then subsequently stored at −80°C before processing to evaluate the following markers of intestinal health: mucin2 (MUC2), claudin-1 (CDLN1), and occludin (OCNL) mRNA expression.

Macroscopic (duodenum, jejunum and ileum) and histological (ileum) intestinal alterations were evaluated using the I See Inside methodology (ISI) according to Kraieski et al. (18) and Belote et al. (19) at 21 days post-hatch. In the ISI methodology, an impact factor (IF) is defined for each alteration in macroscopic and histologic analysis according to the reduction of organ's functional capacity, based on previous knowledge of literature and background research (18, 19, 22). The IF ranges from 1 to 3, in which 3 is the most impactful alteration for the organ's function (e.g., necrosis, has the highest IF because the functional capacity of affected cells is totally lost). In addition, the extent of each lesion (intensity) or observed frequency is evaluated in each organ/tissue per animal and the score ranges from 0 to 3: score 0 (absence of lesion), score 1 (alteration up to 25% of the area or observed frequency), score 2 (alteration ranges from 25 to 50% of the area or observed frequency), and score 3 (alteration extent more than 50% of the area or observed frequency). To obtain the final value of the ISI index referred as ISI total score, the IF of each alteration is multiplied by the respective score number and the results of all alterations are summed according to the formula ISI = Σ(IF*S), where IF = impact factor and S = Score. For histological alterations, the same calculation was applied. The ISI scale ranges from 0 to 102 for the macroscopic and 0 to 45 for the histologic analysis.

The macroscopic analysis was evaluated in three birds per pen (*n* = 120) by the same observer. For the histological analysis, samples of the proximal ileum from two birds per pen (*n* = 80) were collected and fixed in Davidson's solution (100 mL glacial acetic acid, 300 mL 95% ethyl alcohol, 200 mL 10% neutral buffered formalin and 300 mL distilled water) for at least 24 h. Then, samples were dehydrated, infiltrated and embedded in paraffin following common histological routine. Blocks were cut in 5 μm sections and stained with hematoxylin and eosin plus Alcian Blue (23). An average of 20 intestinal villi per slide was evaluated per bird in 10× objective (using 20× and 40× objective to confirm alterations) of an optical microscope (Nikon Eclipse E200, São Paulo, SP, Brazil).

### Chemical Analysis and Calculations

Dry matter (DM) analysis of samples was performed after oven drying the samples at 105°C for 16 h [method 934.01; (24)]. The samples of ileal digesta and diets were analyzed for gross energy using a calorimeter calibrated with benzoic acid as a standard (IKA Werke, Parr Instruments, Staufen, Germany). Calculation of ileal digestible energy (IDE) was done afterwards. Crude protein (N × 6.25) was determined by the combustion method (method 968.06; 24). Acid insoluble ash concentration in the diets and ileum samples was determined using the method described by Vogtmann et al. (25) and Choct and Annison (26).

Apparent ileal digestibility and ileal digestible energy (IDE) were calculated using the following equations (27, 28)

$$\begin{array}{l} \text{Digestibility }\left(\%\right)\text{ = }\left[\text{1}-\left(\frac{\text{Mi}}{\text{Mo}}\right)\;\times \;\left(\frac{\text{E0}}{\text{Ei}}\right)\;\right]\;\times \text{100},\\ \text{ IDE }(\text{kcal/kg})\text{ = GEi}-\left[\text{GEo }\times \;\left(\frac{\text{Mi}}{\text{Mo}}\right)\right], \end{array}$$

where M_i_ represents the concentration of acid insoluble ash in the diet in grams per kilogram of DM; M_o_ represents the concentration of acid insoluble ash in the ileal digesta in grams per kilogram of DM output; E_i_ represents the concentration of DM or N in the diet in milligrams per kilogram of DM; and E_o_ represents the concentration of DM or N in the ileal digesta in milligrams per kilogram of DM. GE_i_ is gross energy (kcal/kg) in the diet; GE_o_ is the gross energy (kcal/kg) in the ileal digesta in g/kg DM.

### Isolation and Quantification of Total mRNA

Mucin2 was used as a marker for intestinal barrier. The other genes, CLDN1 and OCLN were used as markers for regulation of the tight junction paracellular permeability barrier. The primer sequences used are listed in Table 2. Total RNA was extracted from the intestinal mucosa and homogenized in Trizol (Invitrogen, California, USA) according to the manufacturer's protocol. The RNA was re-suspended in nuclease-free ultrapure distilled water (Invitrogen, California, USA). Total RNA present in the samples was visualized in 1.5% agarose gel and quantified in NanoDrop ND-2000 (Thermo Fisher Scientific, Massachusetts, USA) and the ratios 260A/280A and 260A/230A were analyzed to confirm the quality of the extraction. The residual DNA was removed by DNase I, amplification grade (Invitrogen, California, USA).

**Table 2:** Primers used for real-time PCR^a^.

**Gene**	**Primer (5^′^-3^′^)**
GAPDH (forward)	TGTGACTTCAATGGTGACAGC
GAPDH (reverse)	GCTATATCCAAACTCATTGTCATACC
MUC2 (forward)	ATGCGATGTTAACACAGGACTC
MUC2 (reverse)	GTGGAGCACAGCAGACTTTG
CLDN1 (forward)	TCTTCATCATTGCAGGTCTGT
CLDN 1 (reverse)	ACTCAAATCTGGTGTTAACGGG
OCLN (forward)	GCTCTGCCTCATCTGCTTCT
OCLN (reverse)	TTCTTCACCCACTCCTCCAC

a *GAPDH, glyceraldehyde-3-phosphate dehydrogenase; MUC2, mucin-2; CLDN1, claudin-1; OCLN, occludin*.

### Quantitative Real-Time PCR

Total RNA (1 μg) was reverse-transcribed onto cDNA using iScript cDNA synthesis kit (BIO-RAD Hercules, California, USA). The Real-time PCR (RT-qPCR) was conducted in Quant studio 3 system. For each reaction, the 20 μL mixture contained 5 ng of cDNA, 1× PCR buffer, 2 mM of MgCl_2_, 0.2 μm each of the forward and reverse primers, 0.1X of SYBR green (Invitrogen, California, USA), 0.2 mM of deoxyribonucleotide triphosphate (Invitrogen, California, USA), and 0.25 U taq of Platinum Taq DNA Polymerase kit (Thermo Fisher Scientific, Massachusetts, USA). The amplification protocol was as it follows: 94°C for 5 min (1 cycle), followed by 40 cycles at 94°C for 15 s, 60°C for 10 s, and 72°C for 30 s (fluorescence collection). After the amplification step, a thermal denaturing cycle was included to obtain the dissociation curve of the PCR products to verify the amplification specificity. A negative control sample without any cDNA was included in all plates. All analyses were performed with technical duplicates and two to 10 biological replicates. Relative target gene expression level was determined by the comparative cycle threshold (CT) method (29). Glyceraldehyde-3-phosphate dehydrogenase (GAPDH) gene was used as housekeeping control to normalize variations in the mRNA amount for the target genes.

### Statistical Analysis

The parametric data were subjected to analysis of variance (ANOVA) using the GLM procedure of SAS Institute (30). Means were analyzed by Fisher LSD. The non-parametric data were submitted to the Kruskal-Wallis. Significance was accepted at *P* < 0.05 and tendency at *P* < 0.10.

## Results

There was no effect of treatment on broiler mortality (overall average was 2%; Table 3). From 1 to 21 days, the non-challenged group had the greatest BWG (*P* < 0.001) while challenged birds supplemented with AGP or P(OA+EO) could alleviate the negative effects of the challenge and had greater BWG (*P* < 0.001) than the challenged control group. For the overall period, birds on P(OA+EO) treatment had similar BWG to the non-challenged control group, which was higher than the challenged control and AGP groups (*P* < 0.001). Worse FCR (*P* < 0.001) was observed for the challenged control group compared to all other treatments from 1 to 21 days and 1 to 42 days. The FCR of birds on P(OA+EO) treatment was not different from the non-challenged group from 1 to 21. Cumulative FCR from 1 to 42 days was better (*P* < 0.001) when broilers were not challenged or fed diets containing AGP or P(OA+EO) compared to the challenged control group. The challenge negatively impacted feed intake, which was fully reverted by the supplementation with P(OA+EO) from 21 to 35 days (*P* < 0.001) and partially reverted from 1 to 42 days (*P* < 0.05).

**Table 3:** Growth performance of broiler chickens undergoing an intestinal challenge and fed diets supplemented or not with an antibiotic growth promoter (AGP) or a protected blend of organic acids and essential oils [P(OA+EO)]^1^.

**Item**	**Non-challenged control**	**Challenged control^2^**	**Challenged + AGP^3^**	**Challenged + P(OA+EO)^4^**	**SEM**	***P*-value**
**1–7 days**						
FI, g	160	151	153	150	2	0.178
BWG, g	129^a^	116^c^	118^bc^	121^b^	1	<0.001
FCR	1.238	1.299	1.294	1.243	0.015	0.305
**7–21 days**						
FI, g	1,209	1,158	1,174	1,177	8	0.120
BWG, g	965^a^	886^c^	924^b^	943^ab^	6	<0.001
FCR	1.253^a^	1.307^b^	1.271^a^	1.248^a^	0.006	<0.001
**21–35 days**						
FI, g	2,119^a^	2,012^b^	1,987^b^	2,144^a^	17	<0.001
BWG, g	1,350^a^	1,246^c^	1,297^b^	1,337^ab^	10	<0.001
FCR	1.570^ab^	1.615^b^	1.532^a^	1.604^b^	0.009	0.005
**35–42 days**						
FI, g	1,419	1,396	1,361	1,388	13	0.471
BWG, g	854^a^	804^b^	831^ab^	866^a^	8	0.041
FCR	1.661^b^	1.737^c^	1.639^ab^	1.604^a^	0.012	<0.001
**1–21 days**						
FI, g	1,352^a^	1,300^b^	1,310^b^	1,292^b^	8	0.015
BWG, g	1,094^a^	1,003^c^	1,043^b^	1,063^b^	7	<0.001
FCR	1.236^ab^	1.296^c^	1.256^b^	1.215^a^	0.007	<0.001
**1–42 days**						
FI, g	4,649^a^	4,442^b^	4,423^b^	4,529^ab^	26	0.005
BWG, g	3,298^a^	3,053^c^	3,170^b^	3,266^a^	22	<0.001
FCR	1.409^a^	1.455^b^	1.395^a^	1.387^a^	0.006	<0.001
Mortality, %	2.2	1.8	2.6	2.2	0.3	0.913

*FI, Feed intake; BWG, body weight gain; FCR, Feed conversion ratio*.

a,b,c Means with different superscript letter differ (P < 0.05) based on Fisher LSD honestly significant difference test. SEM, Standard error of the mean.

1 *Means were obtained from 10 replicate pens with 27 birds each at the start of the experiment*.

2 *Challenge: coccidiosis vaccine (Bio-Coccivet) at 10× the manufacturer recommendation dose on day 1, and Clostridium perfringens inoculation at 11, 12, and 13 days of age*.

3 *AGP = antibiotic growth promoter, enramycin at 10 g/t*.

4 *P(OA+EO) = Protected organic acids and essential oils at 300 g/t*.

The lowest DM digestibility and IDE (*P* < 0.05; Table 4) were observed for the challenged control group, while challenged birds on AGP or P(OA+EO) presented similar means to the non-challenged control birds. The IDE was improved (*P* < 0.05) by 92 and 106 kcal/kg when AGP or P(OA+EO) were supplemented in challenged birds, respectively. The supplementation with P(OA+EO) provided a 4.9% increase in DM digestibility when compared to challenged control birds (*P* < 0.01). The ileal digestibility of N was not different among treatments (*P* > 0.05).

**Table 4:** Serum FITC-d and apparent ileal digestibility of broiler chickens undergoing an intestinal challenge and fed diets supplemented or not with an antibiotic growth promoter (AGP) or a protected blend of organic acids and essential oils [P(OA+EO)]^1^.

**Item**	**Non-challenged control**	**Challenged control^2^**	**Challenged + AGP^3^**	**Challenged + P(OA+EO)^4^**	**SEM**	***P*-value**
**Intestinal integrity, 17 days**						
FITC-d, μg/mL	0.169^bc^	0.191^c^	0.148^ab^	0.142^a^	0.005	0.001
**Apparent ileal digestibility, 21 days**						
Dry matter, %	64.2^a^	61.0^b^	63.7^a^	64.3^a^	0.4	0.005
Nitrogen, %	81.6	79.8	81.6	82.0	0.4	0.202
IDE^2^, kcal/kg	3,268^a^	3,143^b^	3,236^a^	3,249^a^	15	0.015

a,b,c *Means with different superscript letter differ (P < 0.05) based on Fisher LSD honestly significant difference test*.

1 *Blood samples were taken from one bird per pen (n = 40). Means of ileal digestibility were obtained from 10 replicate pens of four birds per replicate pen (n = 160)*.

2 *IDE = ileal digestible energy on dry matter basis*.

3 *Challenged: coccidiosis vaccine (Bio-Coccivet) at 10× the manufacturer recommendation dose on day 1, and Clostridium perfringens inoculation at 11, 12, and 13 days of age*.

4 *AGP = antibiotic growth promoter, enramycin at 10 g/t*.

*^5^P(OA+EO) = Protected organic acids and essential oils at 300 g/t*.

Birds on P(OA+EO) group had the lowest serum FITC-d concentration, followed by the AGP group (*P* < 0.05; Table 4). As expected, the challenged control group presented the greatest value.

Broilers on P(OA+EO) group had the lowest total ISI macroscopic score (*P* ≤ 0.05; Table 5) and total ISI histologic score (*P* < 0.05). This result may be attributed to the reduced alterations related to *Eimeria* lesions in the duodenum, inflammatory cell infiltration on epithelium, inflammatory cell infiltration in the lamina propria, lamina propria thickness, and presence of oocysts in the ileum (*P* < 0.05).

**Table 5:** I See Inside (ISI) responses at 21 d of broiler chickens undergoing an intestinal challenge and fed diets supplemented or not with an antibiotic growth promoter (AGP) or a protected blend of organic acids and essential oils [P(OA+EO)]^1^.

**Item**	**Non-challenged control**	**Challenged control^2^**	**Challenged + AGP^3^**	**Challenged + P(OA+EO)^4^**	**SEM**	***P*-value**
**Macroscopic**						
*Eimeria* lesion	0.80^a^	1.67^b^	0.93^a^	0.87^a^	0.12	0.026
ISI total score	12.67^b^	11.63^b^	10.63^ab^	9.34^a^	0.44	0.053
**HISTOLOGIC**						
Inflammatory cell infiltration on epithelium	0.42^a^	0.55^b^	0.42^a^	0.39^a^	0.02	0.001
Inflammatory cell infiltration in the lamina propria	1.61^c^	1.20^b^	1.27^b^	1.01^a^	0.04	<0.001
Presence of oocysts	0.20^b^	0.25^b^	0.40^c^	0.05^a^	0.02	<0.001
Lamina propria thickness	2.29^b^	1.93^a^	2.01^a^	1.91^a^	0.03	<0.001
ISI total score	7.07^b^	6.88^b^	6.81^b^	6.18^a^	0.10	0.016

a,b,c *Means with different superscript letter differ (P < 0.05) based on Fisher LSD honestly significant difference test*.

1 *Macroscopic and microscopic evaluations were obtained from two birds per pen (n = 80)*.

2 *Challenged: coccidiosis vaccine (Bio-Coccivet) at 10× the manufacturer recommendation dose on day 1, and Clostridium perfringens inoculation at 11, 12, and 13 days of age*.

3 *AGP = antibiotic growth promoter, enramycin at 10 g/t*.

4 *P(OA+EO) = Protected organic acids and essential oils at 300 g/t*.

**Figure 1:**
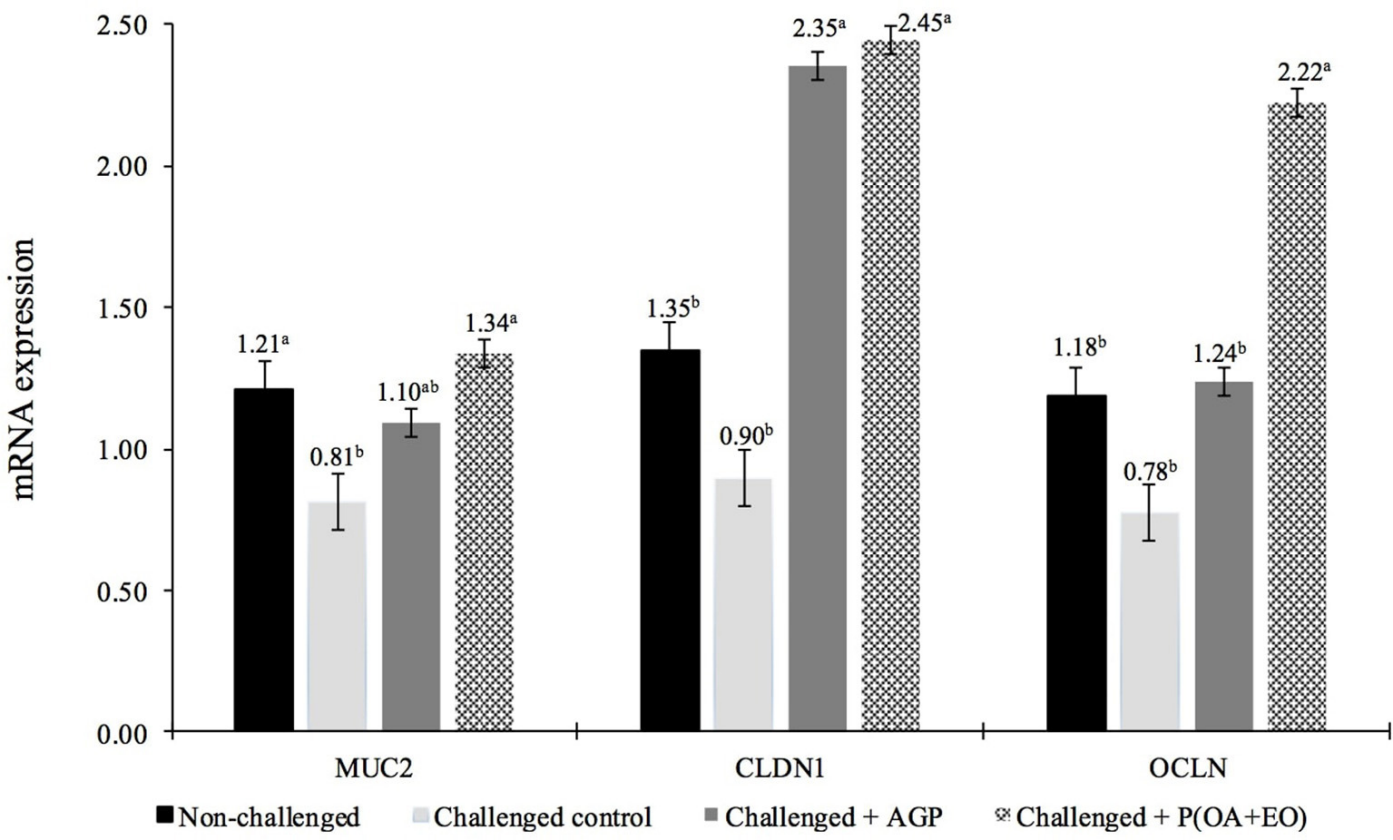
Jejunal gene expression responses at 21 days of broiler chickens undergoing an intestinal challenge and fed diets supplemented or not with an antibiotic growth promoter (AGP) or a protected blend of organic acids and essential oils [P(OA+EO)]. Mucosa samples were taken from one bird per pen (*n* = 60). Evaluated genes were mucin2 (MUC2), claudin1 (CLDN1), and occludin (OCLN). All values are in arbitrary units (bars indicate the SEM; ^a,b^*P* < 0.05 for MUC2 and ^a,b^*P* < 0.001 for CLDN1 and OCLN; Fisher LSD).

At 21 days, non-challenged control and P(OA+EO) groups had the greatest jejunal expression (*P* < 0.05) of MUC2 (Figure 1). Additionally, CLDN1 and OCLN gene expressions were upregulated for the P(OA+EO) birds compared to challenged and non-challenged control groups (*P* < 0.001).

## Discussion

In the present study, coccidial vaccine (at 10× the manufacturer's recommendation dose) and oral gavage with *C. perfringens* were used to induce the experimental intestinal challenge. This challenge model was used to cause a disturbance in the intestinal homeostasis and to allow the evaluation of growth performance, nutrient digestibility, and intestinal health of broilers receiving either AGP or a blend with P(OA+EO) in the feed. The main objective was not to evaluate *Eimeria* infection, once it was not an expected effect of the studied feed additive to act on *Eimeria*, but on dysbiosis caused by this infection. Additionally, the challenge did not result in evidently clinical signs of necrotic enteritis and related mortality.

In order to develop strategies to help broilers reach maximal potential growth performance, it is important to increase our knowledge on the mechanisms involved in the functionality and health of the intestine (31). *Eimeria* spp. and *C. perfringens* challenge are known to damage intestinal mucosa, reducing digestion, and absorption (32). These microorganisms usually cause intestinal inflammation (33), which can reduce FI, increase energy demands (34), and negatively impact FCR (35, 36). Indeed, the intestinal challenge model used in the present study was efficient in reducing growth performance, IDE, and dry matter digestibility. In the overall period, BWG of challenged control group was 7.5% inferior when compared to non-challenged group. Bortoluzzi et al. (37) submitted broilers to a coccidial vaccine challenge and *C. perfringens* using a similar challenge model and observed a 11.2% reduction on BWG from 1 to 42 days. The FCR was 4.6% and 3.2% worse for challenged control group compared to non-challenged group from 1 to 21 days and 1 to 42 days, respectively. These results are also in agreement with Belote et al. (22) who used a similar challenge model and found that FCR was 6.8% worse in challenged compared to non-challenged birds from 1 to 21 days. Additionally, in the present study, the challenge model reduced DM digestibility by 2.2% and IDE by 124 kcal/kg. The poor growth performance and reduced nutrient digestibility observed for the challenged control group in the present trial could be a result of an inflammatory process and intestinal damage, as challenged broilers had higher ISI scores for inflammatory cell infiltration on epithelium and *Eimeria* lesions.

The ISI total score, however, did not show a significant difference among control groups, mainly due to a great inflammatory cell infiltration in the lamina propria and increased lamina propria thickness in the non-challenged control group. The serum FITC-d concentration and CLDN1 and OCLN mRNA expressions were also statistically the same among control groups and the samples were collected at the same age as the samples for the ISI analysis (21 days). Claudin-1 and OCLN are markers for regulation of the tight junction paracellular permeability barrier. These tight junctions help to seal the space between two enterocytes avoiding the translocation of microorganisms, toxins, or any other harmful molecule from the intestinal lumen into the bloodstream. Any damage to the tight junction's functionality could increase intestinal permeability (38), as observed in the present study for the control groups. One may hypothesize that the intestinal mucosa of the non-challenged control chickens was unprotected, since they not receive any intestinal protective feed additive and they might had been under a late cross-contamination during the sapling period. Still, despite these results, the better growth performance and digestibility of broilers on the non-challenged control group suggest that direct, high level, and early challenge exposure to the challenge impacted animal health more than the hypothesized cross contamination. Belote et al. (19) showed that damage in the epithelium at 14 days (higher ISI score) has great correlation with worse FCR up to 42 days.

In the current study, challenged broilers fed diets supplemented with AGP had improved BWG, FCR, digestibility, and intestinal integrity compared to challenged control broilers. However, the ISI total histologic score was not statically different from the challenged control group, mainly due to a great presence of oocysts and similar score for inflammatory cell infiltration in the lamina propria and lamina propria thickness. The beneficial effects on growth performance of broilers receiving AGP are considered clear (39), although the exact mode of action of this additive remains unclear. Possible mechanisms by which AGP impact growth rate and feed efficiency are reducing the severity of intestinal diseases, stabilizing microbial populations and reducing inflammatory response as well as the suppression of bacterial pathogens in the GIT (39, 40). However, with the restriction of antibiotic growth promoters in poultry production systems, there is a growing concern about the crescent incidence of intestinal diseases. Consequently, investing in studies in this field is indispensable to develop strategies to face this present challenge for the industry.

In this context, OA have been supplemented in poultry diets all over the world. They are involved in the inhibition of pathogens present in the intestinal environment and they have an important role in the development and reparation of intestinal wall (41, 42). Yang et al. (43) report that OA act to improve broiler growth performance through different modes of action, such as improving digestive functions and stimulating the growth of beneficial bacteria. Also reported to promote benefits, EO have been described to stimulate digestion and feed intake, to have antioxidant activity, antimicrobial properties, and to increase pancreatic secretion (41, 44, 45). Therefore, in order to take advantage of the synergistic effects of supplementing AO and EO blends, there has been an increase interest of the poultry industry in the use of these additives (46). Organic acids need to cross the bacterial cell membrane to alter its metabolism. As one of the effects of EO is to damage the bacterial cell membrane (47), allowing a greater amount of organic acids to penetrate into the bacterial cytoplasm. The OA, under the undissociated form, are able to reduce the intracellular pH and disturb the bacterial metabolism causing the death of pH sensitive bacteria, such as *E. coli, Salmonella*, and *C. perfringens*. This mechanism of action characterizes the synergic antibacterial effect of the OA and EO (48, 49).

However, besides the blend combination and composition, a special attention is necessary to the product offering form. Organic acids and EO can be easily absorbed in the duodenum, limiting its effectiveness throughout the intestine (50). In addition, OA must be under the undissociated form to cross the bacterial membrane and only when in contact with the higher pH of the bacterial cytoplasm, release H^+^ ions and promote their antimicrobial effect. The large pH variation throughout the bird's GIT may induce the dissociation of the OA prior to the contact with the target bacteria. This is especially important because the main site of interest for modulation of the microbiota is the final portion of the GIT, where the potential pathogenic bacteria are present in the greatest amount. For this reason, it is important that blends of OA and EO are protected with a technology that allows the gradual release of these active compounds along the entire GIT targeting the distal part of the GIT, prevents their rapid degradation in the upper segments, avoids the interaction with other components of the diet and do not interfere with palatability (51, 52) improving its efficiency for poultry (11).

In the present study, challenged broilers fed diets supplemented with a blend of P(OA+EO) had an improvement on BWG (6.5%) and FCR (4.7%) compared to the challenged control group for the overall period. Additionally, this improvement on broilers' growth performance can be partially explained by the greater ileal digestibility observed at 21 days. Ileal digestibility of dry matter and IDE increased 3.2% and 106 kcal/kg, respectively, when broilers were fed diets supplemented with P(OA+EO) compared to the challenged control group. The great digestibility can be related to a better morphological intestinal mucosa and/or a better intestinal digestive activity. Yang et al. (43) found increased villus height and villus height to crypt depth ratio in the jejunum of broilers fed diets supplemented with OA, which is in agreement with the improvement on absorption efficiency observed at 21 days in the present study. Additionally, EO have been proven to stimulate the activity of digestive enzymes and improving nutrient digestibility (53). Liu et al. (11) reported an improvement of trypsin, chymotrypsin and lipase in broilers fed a diet supplemented with a blend of protected OA and EO, similar to the present study. Using the same blend of P(OA+EO) as in the present study, Moraes et al. (54) found that when combining the blend with an AGP (enramycin), there was an improvement on dry matter, crude protein, and energy coefficients of digestibility compared to when AGP was supplemented by itself.

The better growth performance and digestibility could be also associated with a better overall intestinal health status observed for the P(OA+EO) group. For example, in the present study the blend of P(OA+EO) was effective in protecting the intestinal mucosa. In addition to being essential for digestion and absorption processes, the intestinal mucosa is also responsible for protecting the animal against microbial infection. The FITC-d serum concentration at 17 days observed in challenged broilers can be used as an intestinal integrity parameter. Damage caused to tight junction proteins functionality can lead to increased intestinal permeability. Thus, after oral administration a greater amount of FITC-d can cross the intestinal lumen and be found in the bloodstream (55). Increased serum FITC-d levels indicate damage to the intestinal mucosa. In the present study, FITC-d blood levels were lower for broilers on P(OA+EO) group, indicating that even being challenged with the experimental model, their intestinal barriers seemed to be intact and less compromised, suggesting an appropriate intestinal permeability with lower inflammatory-associated enteric epithelial leakage. Additionally, the relative mRNA expression of the tight junction protein, CLDN1, was upregulated in broilers on AGP and P(OA+EO) groups compared to challenged or non-challenged groups. As the main action of CLDN-1 is to maintain the integrity of intestinal barrier, its upregulation may be associated with the better intestinal integrity and lower permeability that were shown to be improved in these groups. The expression of OCLN also was upregulated in broilers fed P(OA+EO) compared to all other treatments. The upregulation of OCLN and CDLN1 in broilers supplemented with EO was previously reporter by Du et al. (56).

Besides tight junction proteins, mucins produced by Goblet cells form the first line of defense in maintaining intestinal barrier (57). Mucin-2 is the main mucin produced and is considered a biomarker of intestinal health because it avoids microbial adhesion to the mucosa (58, 59). In the present study, broilers on challenged control group had the MUC2 expression downregulated compared to broilers on non-challenged and P(OA+EO) groups. Previous studies have shown that *C. perfringens* or the co-infection with *Eimeria* are effective in reducing the expression of MUC2 (60), which decreases the intestinal protection. According to Forder et al. (60), a possible explanation for the reduction of MUC2 in animals challenged with *Eimeria/C. Perfringens* would be the lower capacity for mucosal renewal due to the damage caused by the challenge. The reduced ISI scores observed in broilers fed P(OA+EO) in the present study can be an indication of a lower demand for mucosal renovation (better overall intestinal health), which may explain the greater expression of MUC2 in this treatment. In addition, the lowest ISI total scores observed for the P(OA+EO) group, supported by the lower inflammation in the lamina propria and lower presence of oocysts, are in accordance with the lower damage on the mucosa in response to the intestinal challenge applied.

The positive results observed for the P(OA+EO) supplemented birds are in accordance with other studies reported in the literature (11, 46, 50). The improved growth performance and intestinal health observed in the current study is in agreement with Liu et al. (11). These authors fed broilers with a protected blend of OA and EO and observed improvements on growth performance, intestinal morphology, and digestive enzyme activities, presenting similar results to enramycin on some intestinal microbes. Therefore, Liu et al. (11) also concluded that the supplementation with this protected blend of OA and EO could be used by the poultry industry as an AGP alternative.

## Conclusion

The challenge model used in the present study was efficient to cause a disturbance in the intestinal homeostasis negatively affecting growth performance, nutrient digestibility, and intestinal health of broiler chickens. The protected blend of organic acids and essential oils evaluated showed improved or similar responses to an AGP in neutralizing the negative effects caused by the experimental challenge model. Thus, this natural product may be used as part of a combined solution for AGP free programs in commercial broiler chicken production.

## Data Availability Statement

The datasets generated for this study are available on request to the corresponding author.

## Ethics Statement

This animal study was reviewed and approved by Ethics and Research Committee of the Federal University of Santa Maria, Santa Maria, Brazil (No. 5404280717).

## Author Contributions

CS: draft and revision of the protocol, *in vivo* trial, data analysis, trial conduction, draft and revision of the manuscript, and final approval for paper publication. DR: *in vivo* trial, data analysis, and FITC-d analysis. YD: digestibility and PCR analyses. AS: PCR analysis, PCR orientation, and calculations. MV and MM: draft and revision of the protocol, interpretation of results, draft and revision of the manuscript, and final approval for paper publication. ES: I See Inside methodology analysis and final approval for paper publication.

### Conflict of Interest

The authors declare that this study received funding from Jefo Nutrition Inc. The funder had the following involvement with the study: draft and revision of the protocol, interpretation of results, draft and revision of manuscript, I See Inside methodology analysis and final approval for paper. MV, MM, and ES are employed by Jefo Nutrition Inc. The remaining authors declare that the research was conducted in the absence of any commercial or financial relationships that could be construed as a potential conflict of interest.
